# Preoperative Embolization and Total Left Pneumonectomy for a Giant Pulmonary Solitary Fibrous Tumor

**DOI:** 10.1016/j.atssr.2025.09.017

**Published:** 2025-10-16

**Authors:** Eitetsu Koh, Yasuo Sekine, Tadao Nakazawa, Kenzo Hiroshima

**Affiliations:** 1 Department of Thoracic Surgery, Tokyo Women's Medical University Yachiyo Medical Center, Chiba, Japan; 2 Department of Pathology, Tokyo Women's Medical University Yachiyo Medical Center, Chiba, Japan

## Abstract

Solitary fibrous tumor (SFT) is a rare mesenchymal neoplasm; pulmonary cases can become massive and hypervascular, increasing surgical risk. A 71-year-old woman with a giant pulmonary SFT underwent preoperative angiography, which identified the internal thoracic artery as the dominant feeder. Selective embolization was performed, followed by total left pneumonectomy without cardiopulmonary support. Pathology confirmed margin-negative (R0) resection without pulmonary infiltration or nodal involvement. The postoperative course was uneventful, and no recurrence has been observed for 2.5 years. Preoperative angiographic evaluation and embolization are effective strategies to enhance surgical safety in managing hypervascular giant pulmonary SFTs.

Solitary fibrous tumors (SFTs) are rare mesenchymal tumors that originate most commonly from the pleura; pulmonary involvement is uncommon. Although typically slow-growing, giant tumors may cause compressive symptoms and carry risks during surgical resection due to hypervascularity. Complete resection is the standard treatment, and preoperative embolization has been reported to reduce intraoperative bleeding.^1^, ^2^, ^3^ However, detailed preoperative vascular evaluation specific to pulmonary SFTs remains limited. We present a case of a giant hypervascular pulmonary SFT resected successfully after preoperative embolization guided by angiographic evaluation.

A 71-year-old woman was referred for evaluation of a large left thoracic mass. Five years earlier, a 4-cm anterior mediastinal lesion had been detected on routine imaging but left untreated (Figure 1A). On reevaluation after cerebral infarction, chest radiograph revealed a massive thoracic shadow (Figure 1B). She was asymptomatic, with acceptable pulmonary function (forced vital capacity, 1.26 L [57.5%], FEV_1_, 1.01 L [80.2%]) and normal cardiac function. Serum tumor markers—including carcinoembryonic antigen, cytokeratin 19 fragment, pro-gastrin-releasing peptide, squamous cell carcinoma antigen, and neuron-specific enolase—were within normal limits.

**Figure 1:**
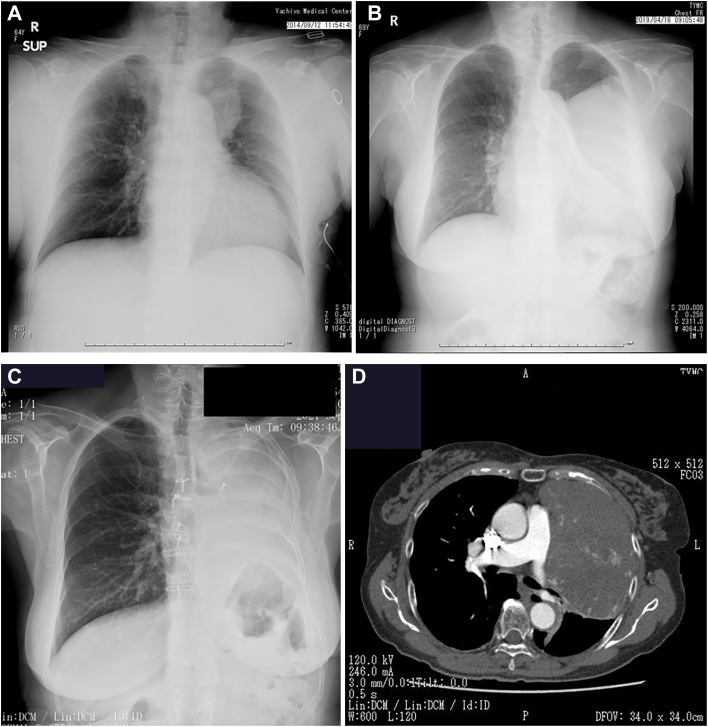
Chest radiographs and computed tomography. (A) Initial chest radiograph showing an anterior mediastinal lesion on earlier evaluation. (B) Chest radiograph on current presentation showing a massive left thoracic opacity with mediastinal shift. (C) Postoperative chest radiograph following left pneumonectomy. (D) Axial contrast-enhanced computed tomography demonstrating a large mass occupying the left hemithorax with mediastinal displacement.

Interleukin-6 and insulin-like growth factor-2 were not assessed. Contrast-enhanced computed tomography revealed a 15-cm mass occupying the left hemithorax with mediastinal shift (Figure 1D). No signs of invasion were observed. Computed tomography-guided biopsy confirmed solitary fibrous tumor. Immunohistochemistry was positive for CD34, STAT6, and BCL2. Given the tumor’s size and suspected hypervascularity, preoperative angiography was performed (Figure 2A). It revealed the left internal thoracic artery, specifically the pericardiophrenic branch, as the main feeder. No significant supply from intercostal or lateral thoracic arteries was noted, suggesting low risk of chest wall invasion. Selective embolization was carried out using gelatin sponge and coil embolization. Postembolization angiography demonstrated marked reduction of tumor blush (Figure 2B).

**Figure 2:**
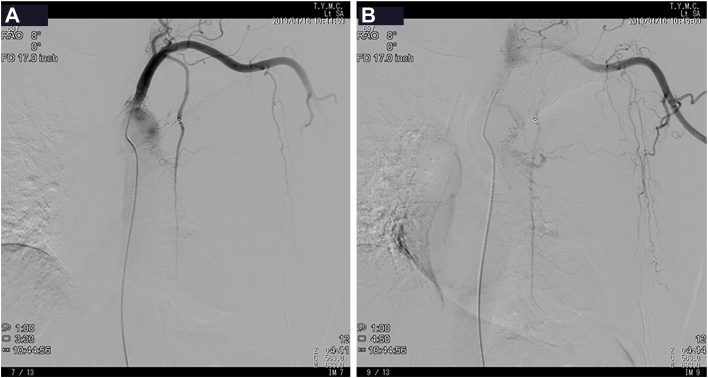
Angiography. (A) Selective angiography of the left internal thoracic artery/pericardiophrenic branch demonstrating prominent tumor blush before embolization. (B) Postembolization angiography showing marked reduction of tumor blush.

Surgery was performed via median sternotomy with lateral extension. Cardiopulmonary bypass was on standby but not used. The tumor was mobilized without chest wall invasion, and total left pneumonectomy was completed (Figure 3A). Blood loss was 872 mL, and no transfusion was required. Histopathology confirmed a solitary fibrous tumor without malignant features (Figure 3B). Surgical margins were clear, and no lymph node metastasis was identified (pN0). The postoperative course was uneventful; a postoperative chest radiograph is shown (Figure 1C). The patient was discharged on day 10. At 2.5 years postoperatively, there is no evidence of recurrence.

**Figure 3:**
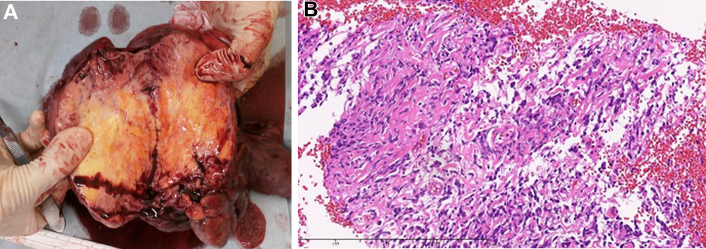
Gross specimen and histology. (A) Gross photograph of the resected specimen. (B) Hematoxylin and eosin–stained section showing spindle cells in a patternless arrangement with staghorn-type vasculature; arrowheads indicate representative spindle cells (original magnification ×100; 10× objective]). Immunohistochemistry, not shown, demonstrated diffuse nuclear STAT6 expression and CD34 positivity.

## Comment

This case illustrates the usefulness of selective preoperative embolization in managing giant hypervascular pulmonary SFTs. Intraoperative bleeding is a major risk in such cases, and prior reports have shown benefits of embolization in controlling hemorrhage.^1^^,^^2^ Our angiographic assessment enabled selective embolization of deep internal thoracic branches, reducing the need for circulatory arrest or blood transfusion. Additionally, angiography confirmed the absence of chest wall supply, avoiding unnecessary resection. Tumor doubling time (410 days) reflected the indolent nature of SFTs, but the risk of bleeding necessitated embolization. Evidence from mediastinal hypervascular tumors also supports preoperative embolization as part of surgical planning,^3^ and our experience suggests that targeted vascular mapping can improve safety and outcomes in giant pulmonary SFTs.

## References

[1] Aydemir B., Çelik S, Okay T, Doğusoy I. (2013). A case of giant intrathoracic solitary fibrous tumor managed by preoperative embolization and surgery. Am J Case Rep.

[2] Weiss B., Horton D.A. (2002). Preoperative embolization of a massive solitary fibrous tumor of the pleura. Ann Thorac Surg.

[3] Lucarelli N.M., Maggialetti N., Marulli G. (2024). Preoperative embolization in the management of giant thoracic tumors: a case series. J Pers Med.

